# Estimating healthcare mobility in the Veterans Affairs Healthcare System

**DOI:** 10.1186/s12913-016-1841-4

**Published:** 2016-10-21

**Authors:** Karen H. Wang, Joseph L. Goulet, Constance M. Carroll, Melissa Skanderson, Samah Fodeh, Joseph Erdos, Julie A. Womack, Erica A. Abel, Harini Bathulapalli, Amy C. Justice, Marcella Nunez-Smith, Cynthia A. Brandt

**Affiliations:** 1 Veterans Affairs Connecticut Healthcare System, West Haven, CT USA; 2 Department of Internal Medicine, Yale School of Medicine, New Haven, CT USA; 3 Yale School of Medicine, Equity Research and Innovation Center, New Haven, CT USA; 4 Department of Psychiatry, Yale School of Medicine, New Haven, CT USA; 5 Yale School of Nursing, West Haven, CT USA; 6 Yale School of Medicine, Center for Medical Informatics, New Haven, CT USA

**Keywords:** Geographic mobility, Migration, Healthcare utilization, Veterans

## Abstract

**Background:**

Healthcare mobility, defined as healthcare utilization in more than one distinct healthcare system, may have detrimental effects on outcomes of care. We characterized healthcare mobility and associated characteristics among a national sample of Veterans.

**Methods:**

Using the Veterans Health Administration Electronic Health Record, we conducted a retrospective cohort study to quantify healthcare mobility within a four year period. We examined the association between sociodemographic and clinical characteristics and healthcare mobility, and characterized possible temporal and geographic patterns of healthcare mobility.

**Results:**

Approximately nine percent of the sample were healthcare mobile. Younger Veterans, divorced or separated Veterans, and those with hepatitis C virus and psychiatric disorders were more likely to be healthcare mobile. We demonstrated two possible patterns of healthcare mobility, related to specialty care and lifestyle, in which Veterans repeatedly utilized two different healthcare systems.

**Conclusions:**

Healthcare mobility is associated with young age, marital status changes, and also diseases requiring intensive management. This type of mobility may affect disease prevention and management and has implications for healthcare systems that seek to improve population health.

## Background

Approximately 12 % of the adult U.S. population are geographically mobile, defined by the U.S. Census Bureau as at least one residential move in the past year; the majority of this group are ethnic/racial minorities, military families, or the unemployed [1, 2]. Past research has demonstrated the effect of residential histories on health, with environmental exposure to substances affecting a person’s risk of disease [3, 4]. Geographic mobility is a phenomenon that encompasses more than residential mobility, and it may affect health through other mechanisms [5–7]. A small body of research has documented specific groups of people who are repeatedly geographically mobile across small and large geographies and who, as a consequence, utilize healthcare in different healthcare systems [5–9].

We introduce the term “healthcare mobility” as defined by healthcare utilization for preventive care and disease management in different healthcare systems. This concept is distinct from medical tourism, or the use of healthcare services for elective procedures in a travel destination that often provides healthcare at a lower cost. The extent of healthcare mobility across the U.S. is not well described. Previous qualitative studies and literature reviews describe different typologies of mobility for healthcare, related to individual (affordability of services, presence of social support) and contextual factors (availability of services) [5–7, 10, 11]. The studies also describe characteristics of this mobility, such as the distance between where healthcare services are received and how often patients repeatedly visit these different locations. Healthcare mobility may have detrimental effects on outcomes of care, such as delays in care or adherence to care plans [6].

Our ability to quantify this type of healthcare utilization and study its effect on healthcare outcomes is limited by the lack of integration among many healthcare systems. Few healthcare systems span large geographic areas, have integrated electronic health information systems, or have patient-level clinical data that can be shared across the systems [12, 13]. The Veterans Health Administration (VHA) is an ideal system in which to examine healthcare utilization across small and large geographies. The VHA healthcare network spans the United States; VHA cares for low-income and minority Veterans who are more likely to be geographically mobile; Veterans in the VHA can receive care at any site; and it has an integrated electronic health record (EHR) with standardized systems. Using data from Veterans who utilize the VHA, we explore the extent of mobility for outpatient healthcare utilization, characterize those individuals who use different healthcare systems, and describe potential mobility patterns for healthcare utilization.

## Methods

### Data source and sample

The VHA is a national, closed, integrated system for healthcare delivery for Veterans. It is comprised of hospitals and community-based outpatient clinics across the United States and its territories. Each hospital is connected with one or more outpatient clinics; together, they are considered a “healthcare system”. In the VHA, there are approximately 152 healthcare systems of which most have individual implementations of the electronic health record (EHR). Providers have easy access to clinical information on patients within their own healthcare system, but must search for patient data from other VHA healthcare systems. Though each of these healthcare systems is operationally managed through one of the nineteen Veteran Integrated Service Network (VISNs), this organizational structure does not affect providers access to Veterans’ EHR data in these healthcare systems.

Much of the VHA clinical and administrative data in the EHR have been systematically organized in the national VHA Corporate Data Warehouse (CDW). The CDW contains historical data beginning in fiscal year (FY) 1999 (October 1998-September 1999), and data are updated daily. Data extracted from the CDW contain demographic and clinical characteristics and healthcare utilization [14, 15].

For this study, we used EHR data from the Musculoskeletal Disorder (MSD) cohort, a previously characterized cohort with over five million Veterans who accessed the VHA between January 1, 2000 and December 31, 2013, and who had an MSD diagnosis as defined by one inpatient or two outpatient MSD diagnoses in an 18 month period [16]. Our sample of interest was Veterans who had at least two outpatient visits within a four year period: from January 1, 2010- December 31, 2013. We used four years of data to capture healthcare utilization patterns over time.

### Variables of interest

The primary dependent variable of interest was healthcare mobility. We defined healthcare mobility as a Veteran’s use of at least two different healthcare systems (heretofore referred to as facility) for any outpatient visit in this four year period, regardless of residential mobility. We defined the levels of healthcare mobility as 1 (used only one facility, or not healthcare mobile), 2 (used two facilities), 3 (used three facilities), and 4 (used four or more facilities).

At the patient-level, we included demographic and clinical characteristics as variables of interest based on prior studies [17–19]. For demographic characteristics, we included age, sex, ethnicity/race, marital status, and Operation Enduring Freedom/Operation Iraqi Freedom (OEF/OIF) status. For clinical characteristics, we included the presence of common chronic conditions in this cohort, including hypertension, coronary artery disease, diabetes, chronic obstructive pulmonary disease, hepatitis C virus infection (HCV), and mental health diagnoses, such as post-traumatic stress disorder (PTSD) and depression. These diagnoses were identified using ICD-9 codes [16]. We defined a Veteran’s primary outpatient facility as the facility where the MSD diagnosis occurred. We included a VHA-defined complexity level for the Veteran’s primary facility [20]. The complexity level is based on a weighted score of several criteria, including the volume and patient case mix, clinical services at a facility, and the number of specialized clinical services (e.g. spinal cord injury, blind rehabilitation, cardiac surgery, interventional cardiac catheterization lab, neurosurgery, transplant, and radiation oncology, and mental health case management program). Complexity levels for all facilities are 1a (most complex), 1b, 1c, 2, and 3 (least complex) [20].

We developed a priori definitions to identify potential healthcare mobility patterns among the healthcare mobile Veterans who had utilized only two facilities. For this sample, we identified two possible types of mutually-exclusive healthcare mobility patterns, such as specialty-care (e.g. individuals who seek specialty care in a location outside of their designated facility) and lifestyle-related (e.g. the sociologic phenomenon of “snow-birds”) [21, 22]. Our main characteristics used to identify these patterns included the number of times a Veteran alternates between two different facilities, the distance between the two facilities (using the geolocation of the facility’s street address), the complexity of services offered at the two facilities, and the Veterans Integrated Service Network (VISN) designation, which represents large geographic regions by which the VHA organizes its information systems. For specialty-care mobility, we premised that 1) changes between the two facilities occurred more frequently as defined by changes in facilities occurring at least 3 times per year and 2) the two facilities had different complexity of services. For lifestyle-related mobility, we premised that 1) the two facilities were separated by 500 miles or more; 2) the facilities were in different VISN locations; and 3) changes between two facilities occurred no more than biannually.

### Statistical analyses

We first used chi-square tests to compare socio-demographic and clinical characteristics of Veterans, stratified by the levels of healthcare mobility. Second, we performed logistic regressions to explore the association between healthcare mobility (use of two or more facilities versus only one facility because of the small sizes in the higher mobility levels) and the socio-demographic and clinical variables of interest. Third, among healthcare mobile Veterans, we described potential patterns for specialty care-related and lifestyle-related mobility. *P* < 0.05 was considered statistically significant for all comparisons. All analyses were performed using SAS version 9.4 (SAS Institute Inc., Cary, NC, USA). The VA Connecticut Healthcare System’s institutional review board approved this study. The study has a waiver of informed consent and is compliant with the Health Insurance Portability and Accountability Act.

## Results

### Sample characteristics

Our sample consisted of 774,188 Veterans from the MSD cohort (Table 1). Of these, 67,942 (8.7 %) Veterans were healthcare mobile: 7.8 % (*n* = 60,942) used two healthcare facilities, 0.8 % (*n* = 6021) used three facilities, and 0.1 % (*n* = 979) used four or more facilities (Table 1). Women, white, unmarried or divorced, OIF/OEF service Veterans were significantly more likely to be healthcare mobile. Veterans with a mental health diagnosis were also more likely to be healthcare mobile (*p* < 0.0001).

**Table 1:** Socio-demographic and Clinical Characteristics of Veterans by level of healthcare mobility 2010–2013^a^

		*Healthcare mobility levels*								
	*Overall*	*Level 1*		*Level 2*		*Level 3*		*Level 4*		
	*N*	*N*	*(%)*	*N*	*(%)*	*N*	*(%)*	*N*	*(%)*	*p-Value*
	774,188	706,246	(91.2)	60,942	(7.8)	6,021	(0.8)	979	(0.1)	
*Sex*										<0.0001
*Female*	52,017	45,612	(6.5)	5,613	(9.2)	686	(11.4)	106	(10.8)	
*Male*	722,171	660,634	(93.5)	55,329	(90.8)	5,335	(88.6)	873	(89.2)	
*Age*										<0.0001
*18*–*25*	22,402	19,530	(2.8)	2,500	(4.1)	318	(5.3)	54	(5.5)	
*25*–*40*	92,342	81,066	(11.5)	9,840	(16.1)	1,184	(19.7)	252	(25.7)	
*41*–*50*	123,632	108,069	(15.3)	13,352	(21.9)	1,856	(30.8)	355	(36.3)	
*51*–*60*	205,488	184,163	(26.1)	19,275	(31.6)	1,805	(30.0)	245	(25.0)	
*61*–*70*	174,352	163,703	(23.2)	9,977	(16.4)	616	(10.2)	56	(5.7)	
*71*–*80*	113,469	108,434	(15.4)	4,807	(7.9)	213	(3.5)	15	(1.5)	
*81*	42,503	41,281	(5.8)	1,191	(2.0)	29	(0.5)	2	(0.2)	
*Race/Ethnicity*										<0.0001
*White*	557,744	508,768	(72.0)	44,019	(72.2)	4,287	(71.2)	670	(68.4)	
*Black*	126,089	113,963	(16.1)	10,667	(17.5)	1,221	(20.3)	238	(24.3)	
*Hispanic*	41,189	37,407	(5.3)	3,435	(5.6)	301	(5.0)	46	(4.7)	
*Other*	19,752	17,843	(2.5)	1,724	(2.8)	165	(2.7)	20	(2.0)	
*Unknown*	29,414	28,265	(4.0)	1,097	(1.8)	47	(0.8)	5	(0.5)	
*Marital status*										<0.0001
*Married*	423,299	392,067	(55.5)	28,722	(47.1)	2,259	(37.5)	251	(25.6)	
*Not Married*	88,725	79,868	(11.3)	7,756	(12.7)	931	(15.5)	170	(17.4)	
*Separated/Divorced*	203,148	179,193	(25.4)	20,906	(34.3)	2,528	(42.0)	521	(53.2)	
*Widowed*	56,737	52,901	(7.5)	3,497	(5.7)	302	(5.0)	37	(3.8)	
*Unknown*	2,279	2,217	(0.3)	61	(0.1)	1	(0.0)	0	0	
*OEF/OIF SERVICE*	57,588	51,853	(7.3)	5,069	(8.3)	564	(9.4)	102	(10.4)	<0.0001
*Clinical characteristics* ^*b*^										
*Hypertension*	361,351	334,365	(47.3)	24,597	(40.4)	2,104	(34.9)	285	(29.1)	<.0.0001
*Coronary artery disease*	106,030	98,615	(14.0)	6,812	(11.2)	533	(8.9)	70	(7.2)	<.0.0001
*Diabetes*	140,178	129,632	(18.4)	9,635	(15.8)	803	(13.3)	108	(11.0)	<.00001
*COPD*	54,942	50,263	(7.1)	4,217	(6.9)	403	(6.7)	59	(6.0)	0.0888
*Depressive Disorders*	129,776	122,767	16.0)	14,696	(24.1)	1,913	(31.8)	400	(40.9)	<.00001
*PTSD*	72,488	62,483	(8.8)	8,684	(14.2)	1,096	(18.2)	225	(23.0)	<.00001
*Hepatitis C*	19,952	17,136	(2.4)	2,375	(3.9)	358	(5.9)	83	(8.5)	<.00001
*Digestive system cancer including colon*	4,766,	4,470	(0.6)	280	(0.5)	16	(0.3)	0	0	<0.0001
*Lung cancer*	2,433	2,273	(0.3)	152	(0.2)	7	(0.1)	1	(0.1)	<0.0004
*Facility complexity levels* ^*C*^										<0.0001
*1a*	293,376	270,142	(38.3)	20,958	(34.4)	1,969	(32.7)	307	(31.4)	
*1b*	109,112	100,484	(14.3)	7,657	(12.6)	826	(13.7)	145	(14.8)	
*1c*	162,899	151,979	(21.5)	9,847	(16.2)	897	(14.9)	176	(18.0)	
*2*	118,502	106,486	(15.1)	10,801	(17.7)	1,033	(17.2)	182	(18.6)	
*3*	85,844	73,197	(10.4)	11,241	(18.5)	1,235	(20.5)	164	(16.8)	
*No complexity designation*	4,455	3,958	(0.6)	431	(0.7)	61	1.0	5	(0.5)	

^a^On date of entry into cohort

^b^Most current status

^c^The complexity level is a weighted score based on several criteria, including the volume and patient case mix, clinical services at a facility, and the number of specialized clinical services The complexity was defined from designations made in 2011. There were two healthcare facilities that did not have a facility designation. They offered limited services in the process of restructuring

In our fully adjusted analysis (Table 2), Veterans who were more likely to be healthcare mobile were women, age groups 18–25 or 26–40, separated/divorced or widowed. Veterans with HCV, depressive disorder, PTSD (all *p* < .0.0001) were more likely to be healthcare mobile than those without these diseases. Veterans whose primary facility had a complexity level of 2, 3, or none designated were more likely to be mobile as compared to Veterans whose primary facility was more complex. Older as compared to younger Veterans were less likely to be healthcare mobile. Black and Hispanic Veterans were less likely to be healthcare mobile as compared to white Veterans.

**Table 2:** Factors associated with healthcare mobility among veterans (*N* = 774,188)

	*Unadjusted*			*Model with demographic factors*				*Full model*				
	*Odds ratios*	*95 % Wald confidence limits*		*p-Value*	*Odds ratios*	*95 % Wald confidence limits*		*p-Value*	*Odds ratios*	*95 % Wald confidence limits*		*p-Value*
*Sex – Ref = Male*												
*Female*	1.50	1.46	1.54	<.0001	1.1	1.07	1.13	<.0001	1.08	1.05	1.11	<0.0001
*Age – Ref = 41*–*50*												
*18*–*25*	1.02	0.98	1.07	0.336	1.25	1.2	1.31	<.0001	1.27	1.21	1.33	<0.0001
*25*–*40*	0.97	0.94	0.99	0.009	1.07	1.04	1.1	<.0001	1.08	1.05	1.11	<0.0001
*51*–*60*	0.8	0.79	0.82	<.0001	0.77	0.76	0.79	<.0001	0.76	0.74	0.77	<0.0001
*61*–*70*	0.45	0.44	0.46	<.0001	0.43	0.42	0.44	<.0001	0.44	0.43	0.45	<0.0001
*71*–*80*	0.32	0.31	0.33	<.0001	0.3	0.29	0.31	<.0001	0.32	0.31	0.33	<0.0001
*81 and over*	0.21	0.19	0.22	<.0001	0.19	0.18	0.2	<.0001	0.2	0.19	0.21	<0.0001
*Race – Ref = White*												
*Black*	1.10	1.08	1.12	<.0001	0.84	0.82	0.86	<.0001	0.92	0.90	0.94	<0.0001
*Hispanic*	1.05	1.01	1.08	0.005	0.89	0.86	0.92	<.0001	0.96	0.92	0.99	<0.013
*Other*	1.11	1.05	1.16	<.0001	1.0	0.96	1.05	0.866	0.93	0.88	0.97	0.013
*Unknown*					0.31	0.29	0.33	<.0001	0.32	0.31	0.34	<0.0001
*Marital Status – Ref = Married*												
*Not married*	1.39	1.35	1.42	<.0001	1.04	1.01	1.06	0.007	1.05	1.03	1.08	0.6661
*Separated/Divorced*	1.67	1.64	1.70	<.0001	1.39	1.37	1.42	<.0001	1.38	1.36	1.41	<0.0001
*Widowed*	0.91	0.87	0.94	<.0001	1.34	1.29	1.39	<.0001	1.34	1.29	1.39	<0.0001
*Unknown*	0.35	0.27	0.45	<.0001	0.39	0.3	0.5	<.0001	0.39	0.31	0.51	0.0007
*OEF/OIF*	1.16	1.13	1.19	<.0001	0.73	0.71	0.76	<.0001	0.67	0.65	0.69	<0.0001
*Clinical characteristics*												
*Hypertension*	0.73	0.72	0.74	<.0001					0.96	0.94	0.98	<0.0001
*Coronary Artery Disease*	0.78	0.76	0.81	<.0001					1.1	1.07	1.13	<0.0001
*Diabetes*	0.81	0.80	0.83	<.0001					1.0	0.98	1.03	0.677
*COPD*	0.96	0.93	0.99	0.0275					1.09	1.05	1.12	<0.0001
*Depressive Disorders*	1.75	1.72	1.79	<.0001					1.32	1.29	1.35	<0.0001
*PTSD*	1.77	1.73	1.82	<.0001					1.4	1.37	1.44	<0.0001
*Hepatitis C*	1.73	1.67	1.81	<.0001					1.29	1.23	1.34	<0.0001
*Digestive System Cancer*	0.61	0.55	0.66	<.0001					0.96	0.85	1.08	0.523
*Lung cancer*	0.56	0.49	0.64	<.0001					0.98	0.84	1.16	0.837
*Cancer*												
*Facility Complexity Levels Reference = 1a*												
*1b*	0.99.	0.98	1.01	0.8678					0.98	0.96	1.01	0.19
*1c*	0.87	0.86	0.89	<.0001					0.85	0.83	0.87	<0.0001
*2*	1.21	1.18	1.23	<.0001					1.37	1.34	1.4	<0.0001
*3*	1.74	1.71	1.77	<.0001					2.14	2.09	2.19	<0.0001
*No complexity level*	1.40	1.30	1.50	<.0001					1.59	1.45	1.75	<0.0001
*Designation*												

### Patient-level healthcare mobility patterns

**Fig. 1:**
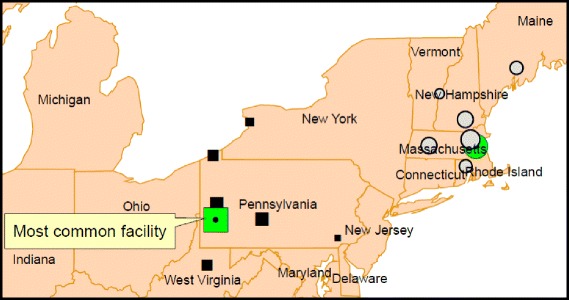
Two most common facilities for specialty care (green square and circle) and their sharing facilities (■/o)

For healthcare mobile Veterans using two facilities (*n* = 60,942), we identified two potential patterns of healthcare mobility using our predefined rules. A total of 12,679 Veterans met our criteria for specialty care mobility Their median rate of changes between the two facilities was 5.8 times per year (range 3–36) with the facilities’ median distance of 95.7 miles. We illustrated the two most frequented facilities for specialty care mobility and the top five facilities that share patients with them (Fig. 1). The most common facility was located in Pennsylvania and had 782 Veterans that were shared with 31 other facilities; the second most common facility was located in Massachusetts and had 738 Veterans who were shared with 23 other facilities.

**Fig. 2:**
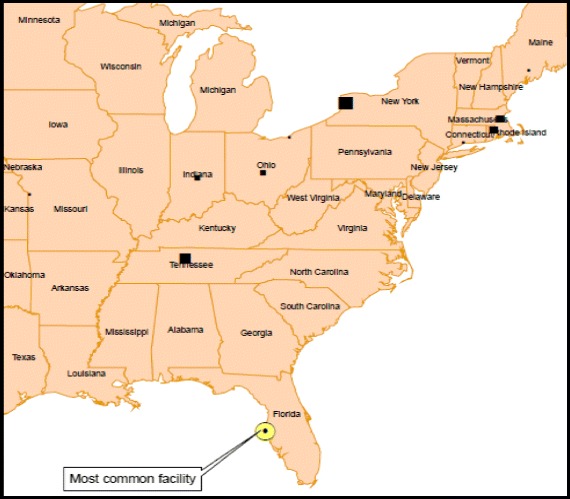
Most common facility used among the lifestyle mobile Veterans (O) and the ten sharing facilities (■)

We also identified 13,502 Veterans who met our criteria for lifestyle-related mobility whose median rate of changes between the two facilities was 0.83 times per year (range 0.2–2) and whose facilities’ median distance was 1038 miles (range 500.2–9316.7). We illustrated the most common facility used among lifestyle mobile Veterans and the top ten facilities that share Veterans with this facility (Fig. 2). This facility was located in Florida and shared 813 Veterans with 110 other facilities.

To provide evidence in support of our algorithms for specialty care and lifestyle mobility, we examined healthcare mobility among Veterans with blindness who used two facilities. Based on our pre-specified algorithm, the proportion of Veterans with blindness (*N* = 191) who are specialty care mobile is 59.1 % (*N* = 113) and those who are lifestyle mobile is 40.8 %. (*N* = 78) (*P* = 0.003). These Veterans are more likely to utilize VHA-designated specialty centers for blind rehab than other VHA facilities. Three of the top five facilities that the specialty care mobile Veterans utilized are VHA-designated specialty facilities for blind rehab. None of the top facilities utilized by lifestyle mobile Veterans are VHA-designated specialty centers for blind rehab.

## Discussion

Within this integrated, national healthcare system, a modest proportion of Veterans are healthcare mobile for outpatient care. Given limited knowledge regarding healthcare mobility, this finding is important because it may affect prevention and management of chronic disease as critical information may reside in different health information systems. Further, we were able to identify different healthcare mobility patterns to demonstrate the temporal and spatial extent of this healthcare mobility.

Our main finding of healthcare mobility for outpatient care among Veterans adds to current knowledge about mobile populations and the extent of mobility across the United States. As this study is a first attempt to systematize the process of exploring mobility among a national sample, we are cautious to draw comparisons between our findings and studies that examined other populations in the United States using different samples and measurements of mobility [23]. Some of our findings are consistent with general geographic mobility trends in the United States [1, 2]. Younger Veterans have greater mobility than other groups possibly due to changes in family, career opportunities and social factors [1, 2]. People who are divorced and separated are also more likely to be healthcare mobile. This mobility may be attributed to residential relocation. Divorce and separation is often associated with high levels of stress which may lead to greater care seeking [24]. Past studies have found that Veterans with mental illness are residentially mobile and have inpatient care in different healthcare systems [5, 6, 17, 18]. Our study builds upon past work demonstrating greater healthcare mobility among Veterans with PTSD and depression. A recent report about homeless Veterans found that 15 % were healthcare mobile within the VHA system [25]. Future work will need to examine the association between homelessness, mental illness and healthcare mobility.

This study also provides a first look at patient-level temporal and geographic patterns of healthcare mobility across the VHA network of facilities. Among Veterans using two facilities, we demonstrated that Veterans are repeatedly accessing outpatient services in different healthcare systems within the VHA. This repeated use of health services in different locations over our study period is not surprising, although it adds a level of complexity to care coordination across care providers and systems. Importantly, we demonstrate that our study population utilizes healthcare in different facilities across both small and large geographic areas and the potential to elucidate different reasons for mobility, such as our example of specialty care and lifestyle mobility patterns. Further characterization will enable us to explore health outcomes and identify specific health needs of distinct mobile populations.

Our study has some limitations to consider. Our study most likely underestimated the extent of healthcare mobility because Veterans use other types of healthcare services with the VHA and use other non-VHA healthcare systems. We know that Veterans use outpatient services paid for by Centers for Medicare and Medicaid Services, with estimates of over 30 % CMS use in specific Veteran populations [26–28]. Although we did not validate healthcare mobility by chart review to document a face-to-face patient-provider encounter at an outpatient facility, we know that telehealth usage for outpatient visits among Veterans is a small proportion of all visits [29]. Lastly, the causality between correlates and our variable of interest cannot be determined. However, we argue that mobility, as a result of or the cause of marital status, for example, may challenge providers and healthcare systems’ ability to coordinate and deliver timely care.

## Conclusion

This study adds to the small body of literature examining healthcare mobility. It is important both to quantify the number of systems each person uses to capture the full extent of care coordination needed and to characterize the heterogeneity among this population. The effect of healthcare mobility on health outcomes are unknown; and therefore, we need to identify subgroups which may be at greater risk for consequences of healthcare fragmentation. In our use case of the VHA, Veterans may utilize healthcare in another location for more timely care or for higher quality of care. For example, in 2014 VHA recently implemented the Veterans Access, Choice and Accountability Act, a policy which allows certain Veterans to utilize healthcare outside of the VHA [30]. Future work includes validating and refining the heterogeneous typologies of mobility, such as the intersection between residential and healthcare mobility, and determining differences in health and healthcare outcomes by these typologies. This work in the context of increasing healthcare options in the United States has implications beyond this Veteran population and for VHA and non-VHA healthcare systems. Current innovations in patient-centered models of care (i.e. primary-care medical homes or accountable care organizations) and information systems (i.e. health information exchanges) will need to account for population mobility across different healthcare systems and geographic borders.

## Abbreviations

CDW: Corporate data warehouse
CMS: Centers for Medicare and Medicaid Services
EHR: Electronic health record
MSD: Musculoskeletal disorder
OEF/OIF: Operation Enduring Freedom/Operation Iraqi Freedom
PTSD: Post-traumatic stress disorder
VHA: Veterans Health Administration
VISN: Veterans Integrated Service Network
